# Physiological Properties of Supragranular Cortical Inhibitory Interneurons Expressing Retrograde Persistent Firing

**DOI:** 10.1155/2015/608141

**Published:** 2015-02-11

**Authors:** Barbara Imbrosci, Angela Neitz, Thomas Mittmann

**Affiliations:** ^1^Institute of Physiology, University Medical Center of the Johannes-Gutenberg University Mainz, 55128 Mainz, Germany; ^2^Neurowissenschaftliches Forschungszentrum, Charité-Universitätsmedizin Berlin, Campus Charité Mitte, Charitéplatz 1, 10117 Berlin, Germany; ^3^Department of Clinical Neurobiology, Medical Faculty of Heidelberg University and German Cancer Research Center (DKFZ), 69120 Heidelberg, Germany

## Abstract

Neurons are polarized functional units. The somatodendritic compartment receives and integrates synaptic inputs while the axon relays relevant synaptic information in form of action potentials (APs) across long distance. Despite this well accepted notion, recent research has shown that, under certain circumstances, the axon can also generate APs independent of synaptic inputs at axonal sites distal from the soma. These ectopic APs travel both toward synaptic terminals and antidromically toward the soma. This unusual form of neuronal communication seems to preferentially occur in cortical inhibitory interneurons following a period of intense neuronal activity and might have profound implications for neuronal information processing. Here we show that trains of ectopically generated APs can be induced in a large portion of neocortical layer 2/3 GABAergic interneurons following a somatic depolarization inducing hundreds of APs. Sparsely occurring ectopic spikes were also observed in a large portion of layer 1 interneurons even in absence of prior somatic depolarization. Remarkably, we found that interneurons which produce ectopic APs display specific membrane and morphological properties significantly different from the remaining GABAergic cells and may therefore represent a functionally unique interneuronal subpopulation.

## 1. Introduction

Neurons are considered polarized functional elements able to receive, to process, and to transmit information unidirectionally. Firstly, synaptic inputs are received and integrated in the somatodendritic compartment. Subsequently, suprathreshold signals trigger action potentials (APs) at the axon initial segment and finally APs are relayed through the axon to the synaptic terminals where they lead to the release of neurotransmitter. Despite these well accepted notions it was recently shown that information, at the level of single neurons, may also travel backward. Specifically, a few studies reported that some neurons in the central nervous system are able, under certain circumstances, to originate ectopic action potentials (APs) in absence of synaptic inputs [1–3]. Ectopic APs are generally originated in axonal segments located distally from the soma and can propagate both orthodromically, toward synaptic terminals, and antidromically toward the soma. This suggests that axons may be not only a relay station but also an independent receptive unit capable of sensing some sort of signals from the surrounding microenvironment and transmitting them both to the postsynaptic target cells and to the integrative element of the parent neuron [3].

Originally ectopically APs were observed in pathological contexts such as in neurons projecting to epileptic foci [1, 2]. However, more recently, ectopic APs have also been observed under physiological conditions suggesting that they may be involved in the physiological functioning of neuronal circuits. In the hippocampus, ectopic APs have been observed in both CA1 [4, 5] and CA3 pyramidal neurons [6] during sharp wave-ripples and high frequency oscillation* in vitro*. Trains of ectopically generated APs were also observed in certain hippocampal and neocortical GABAergic interneurons following natural firing pattern both* in vitro* [7] and* in vivo* [8]. These ectopic APs occurred following hundreds of current injection-induced somatic spikes, outlasted the stimulus by seconds to minutes, and could even be induced in one cell following stimulation of a neighboring interneuron. To describe this form of neuronal activity-induced repetitive ectopic firing observed at GABAergic interneurons, Sheffield and colleges [7] coined the term “persistent firing.” They also suggested that this phenomenon may constitute a previously unknown form of neuronal communication operating on a relatively long time scale which may be capable of conveying information about the recent history of neuronal activity. In the hippocampus this phenomenon has been shown to preferentially occur in interneurons expressing the serotonin 5b receptor [7] and in Ivy interneurons expressing the neuropeptide Y [9]. Herein we have used a complementary approach to further investigate the physiological identity of cortical GABAergic interneurons displaying this specific firing behavior. In cortical layer 2/3 we could trigger persistent ectopic APs in around 30% of interneurons. Furthermore, we also found spontaneous ectopic APs in a large portion of layer 1 interneurons. Interestingly, we observed that persistent firing interneurons possess membrane properties which tend to minimize their recruitment during physiological network activity. In light of this finding we discuss how and which physiological circumstances could induce this unusual mode of firing in this functionally distinct interneuronal class.

## 2. Materials and Methods

### 2.1. Ethical Statement

All experiments were conducted in accordance with EU directive 86/609/EEC for the use of animals in research and the NIH Guide for the Care and Use of Laboratory Animals, and were approved by the local ethical committee (Landesuntersuchungsanstalt RLP, Koblenz, Germany). All efforts were made to minimize the number of animals and their suffering.

### 2.2. Electrophysiology

To identify layer 2/3 interneurons we employed GAD67-GFP heterozygous mice (*n* = 30) initially generated by Tamamaki et al. [10]. Recordings from layer 1 interneurons were performed in C57BL/6 wild type mice (*n* = 8). Mice at the age between p24 and p28 were deeply anaesthetized with isoflurane and decapitated. Coronal slices containing the visual cortex (300 *μ*m) were prepared by use of a vibratome (LEICA, VT-1000-S, Germany). The tissue was incubated at room temperature for 1 hour in a standard Artificial CerebroSpinal Fluid (ACSF) containing (in mM): 125 NaCl, 25 NaHCO_3_, 2.5 KCl, 1.5 MgCl_2_, 2 CaCl_2_, 1.25 NaH_2_PO_4_, and 25 D-glucose (pH 7.4) and bubbled with 95% O_2_ and 5% CO_2_. For recordings slices were transferred into a submerged chamber superfused (perfusion rate: 3.5 mL/min) with an ACSF containing (in mM): 126 NaCl, 25 NaHCO_3_, 3.5 KCl, 1 MgCl_2_, 1 CaCl_2_, 1.25 NaH_2_PO_4_, and 25 D-glucose bubbled with 95% O_2_ and 5% CO_2_. The concentrations of K^+^, Ca^2+^, and Mg^2+^ used in this bathing medium were carefully chosen to match the ionic composition of the brain interstitial fluid measured* in vivo* [11–13]. During all recordings the temperature of the perfusing medium was kept at 33 ± 1°C. The intracellular solution contained (in mM) 140 K-gluconate, 8 KCl, 2 MgCl_2_, 4 Na2-ATP, 0.3 Na2-GTP, 10 Na-phosphocreatine, 10 HEPES, and 0.5% biocytin. The pH was set to 7.3 with KOH. To evaluate membrane and firing properties we applied a series of 1 second lasting square pulses of hyperpolarizing and depolarizing currents through the patch-clamp electrode (at 0.1 Hz). We started by applying a −100 pA current pulse and we gradually increased the magnitude of the injected current by 50 pA for each step. The protocol was executed until we reached a saturation point where cells cease to fire APs. During current injection the membrane potential (Vm) of neurons was set to −70 mV. The electrical signals were recorded with an Axoclamp-2B amplifier (AXON Instrument, USA). Spontaneous excitatory postsynaptic currents (sEPSCs) were recorded in voltage clamp at −60 mV near the reversal potential for GABA_A_ receptors with an Axopatch-200B amplifier (AXON Instrument, USA). Data were filtered at 10 kHz and digitized at 20 kHz using a Digidata-1400 system with PClamp 10 software (Molecular Devices, Sunnyvale, CA, USA). PClamp 10.1 and Matlab software was used for offline analysis. Resting membrane potential (Vm) was measured soon after achieving the whole-cell configuration. To analyze the spike threshold we first measured the maximal Vm slope, considered as the peak of the Vm derivative (max dVm/dt) during the upstroke phase of an action potential. The spike threshold was set at the potential at which the Vm derivative reached 3% of the max dVm/dt [14]. Spike half-width was measured as spike duration at half spike amplitude. sEPSCs were semiautomatically identified with Mini Analysis Software (Synaptosoft, USA) and validated by careful visual inspection. Frequency and amplitude of sEPSCs were measured in each neuron as the median of spontaneous events occurring in a period of 60 sec.

### 2.3. Immunohistochemistry

Slices containing biocytin-filled neurons were fixed overnight with 4% paraformaldehyde and rinsed in PBS. For detection of parvalbumin-positive interneurons some slices were treated for 90 min with PBS containing 10% normal goat serum, 0.2% Triton X-100, and 20% avidin (block A, blocking kit, Vector, USA). Subsequently slices were incubated overnight with the primary antibody rabbit anti-parvalbumin (1 : 1000, Swant, Switzerland) diluted in PBS containing 1% normal goat serum, 0.2% Triton X-100, 20% biotin (block B, blocking kit, Vector, USA). The following day parvalbumin-expressing neurons were visualized by incubating slices for 90 min with Cy5-conjugated goat anti-rabbit (1 : 250, Jackson ImmunoResearch Europe) together with streptavidin-conjugated Cy3 (1 : 250, Jackson ImmunoResearch Europe) for the visualization of biocytin-filled neurons. In slices where parvalbumin expression was not detected the incubation with anti-parvalbumin antibody and the following treatment with Cy5-conjugated goat anti-rabbit were omitted. The size of the neuronal soma was measured semiautomatically with the software Image J (National Institutes of Health, USA).

### 2.4. Statistic

Results are presented as mean ± SEM. The statistical significance of the data was evaluated with the software SPSS. One-way ANOVA and post hoc LSD were applied to compare the three physiologically different layer 2/3 neuronal populations. Unpaired Student* t*-test was applied to compare ectopic and nonectopic layer 1 interneurons.

## 3. Results

### 3.1. Properties of Persistent Firing in Layer 2/3 Interneurons

**Figure 1:**
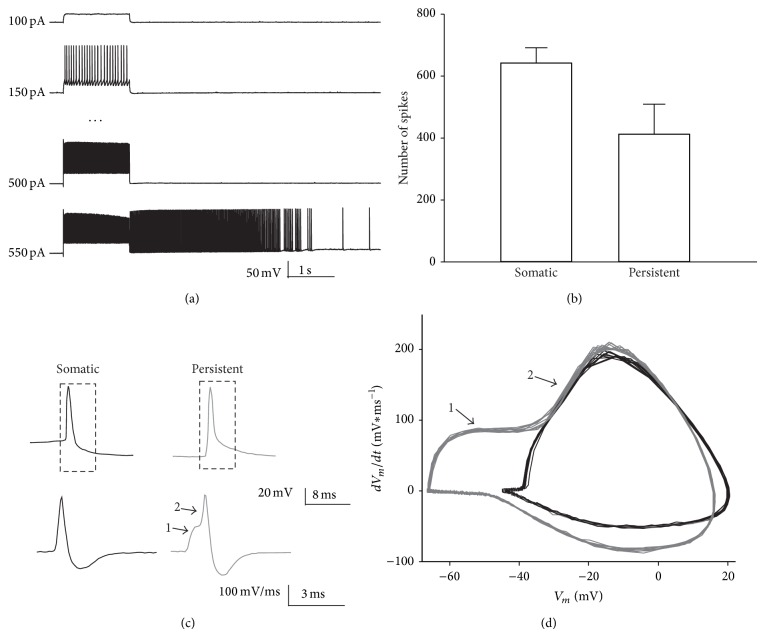
Induction mechanisms and properties of persistent firing in layer 2/3 interneurons. (a) Representative traces showing a series of depolarizing current steps of increasing amplitude (duration of each step: 1 sec) in a current-clamped layer 2/3 interneuron. This protocol eventually led to the generation of persistent firing outlasting the termination of the current step. (b) Graph showing the mean number of current injection-induced somatic spikes needed to evoke persistent firing (somatic) and the mean number of spikes participating in persistent firing (persistent). (c) Spike waveform of one representative somatic and persistent spike in the same neuron (top). The derivative of the rectangular dashed area is represented stretched in time in the bottom traces. Note that the peak of the derivative represents the steepest point of the slope during the spike upstroke; meanwhile at the peak of the spike waveform the value of the derivative is equal to zero. (d) Phase plot showing the rate of change in membrane potential (dVm/dt) as a function of the membrane potential (Vm) for a train of somatic (black traces) and persistent (grey traces) spikes. Note in (c) and (d) the biphasic course of the derivative of the persistent spike. The first component (1) represents the back-propagation of the action potential in the axonal compartment while the second phase (2) represents the spike invasion in the somatodendritic compartment of the neuron. These two phases are better separated and therefore clearly visible only in ectopic spikes due to the long latency for the spike to back-propagate from a distal site in the axon to the somatodentritic compartment of the neuron.

In a substantial portion of interneurons (26.72%, 31 out of 116) repetitive somatic current injections of increasing amplitude (see Section 2) eventually triggered high frequency firing continuing after the termination of the current injection (Figure 1(a)). A form of action potentials-induced persistent firing with similar properties was recently described in hippocampal interneurons [7, 9]. As previously reported, hundreds of action potentials were needed to induce persistent firing (641.89 ± 49.47). The mean number of ectopic spikes generated after the termination of the current injection was 412.42 ± 97.03 (Figure 1(b)) and the median duration 4.12 ± 2.41 sec. A peculiar characteristic of persistent firing was that the participating APs arose abruptly from a very negative membrane potential (generally near resting Vm) without any preceding sign of depolarization [7]. This strictly differed from spikes induced by somatic current injection which only occurred following a strong membrane depolarization (see Table 1 for spike threshold). Persistent APs are believed to originate at distal axonal location and to antidromically propagate toward the soma. The relatively large distance between site of origin and soma may therefore explain the hyperpolarized “apparent” spike threshold which can be measured by the patch-clamp recording electrode only after the somatic invasion of the spike antidromically propagating from distal axonal sites [5]. One way to corroborate the antidromic nature of these spikes could be to perform a collision test by triggering an orthodromic spike (by either somatic current injection or synaptic stimulation) in a very short temporal window after the detection of a spontaneous, presumably antidromic spike. Since the two action potentials will travel through the axon in two opposite directions they will collide and cancel them out at some point in the axon. However, the main limitation of using this approach is that we exclusively performed somatic recordings. Under these recording conditions we can only detect antidromic APs when they are already invading the soma. An alternative possibility to verify a remote site of origin of presumably antidromic spikes is the analysis of their slope. Persistent APs displayed a biphasic course in their slope (Figure 1(c)) which was clearly visible as an inflection in the phase plot of the Vm derivative versus Vm (Figure 1(d)). The first rising phase (1) is thought to represent the spike backpropagation through the axon on the way to the soma, while the second component (2) should represent the spike invasion in the somatodendritic compartment. The clear separation between these two phases is a typical feature of ectopic action potentials due to the long latency for the spike to back-propagate from the axon to the somatodendritic compartment [5] (Figures 1(c)-1(d)).

**Figure 2:**
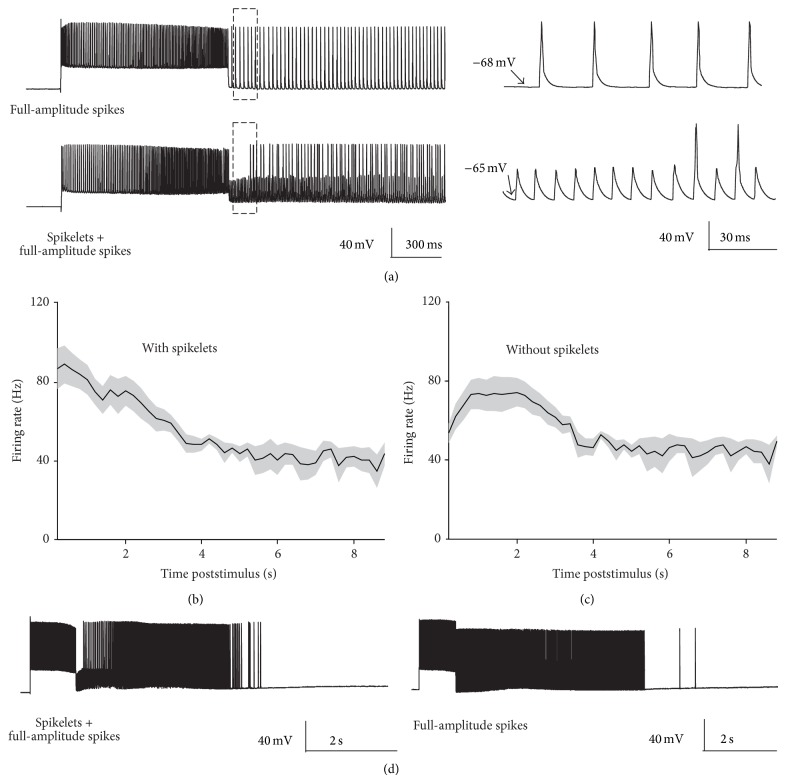
Time course of persistent firing. (a) Persistent spikes consisted of either full size action potentials only (top) or a combination of partial spikes (spikelets) and full size spikes (bottom). The traces on the right represent a magnification of the dashed-rectangle areas to allow a better visualization of both full-amplitude and partial spikes. (b), (c) Frequency of persistent spikes (with or without spikelets, resp.) as a function of time after the termination of the current step. (d) The representative traces show the whole duration of a PF episode from two different neurons. Note the sudden termination of PF at relatively high firing frequency.

Occasionally, during persistent firing we observed ectopic action potentials whose amplitude was roughly half the size of a full-amplitude spike (spikelets) (Figure 2(a), bottom). These spikelets, or partial spikes, have already been observed in hippocampal interneurons and they are believed to represent antidromic APs which fail to invade the somatodendritic compartment of a neuron [7]. Spikelets were mainly present in the initial phase of persistent firing and were gradually substituted by full-amplitude APs (Figure 2(a)). If both full-amplitude and partial spikes were considered, the frequency of persistent firing reached a peak of 89.16 ± 9.59 Hz shortly after the termination of the somatic current injection (Figure 2(b)). When only full-amplitude APs were analysed the frequency of persistent firing reached a slightly lower plateau of 74.55 ± 0.18 Hz at significantly longer latencies (*median latency for peak frequency*, both full-amplitude and partial spikes: 600 ± 178.03 ms, only full-amplitude spike: 1500 ± 156 ms; *P* < 0.05; Figure 2(c)). Following this initial phase the time course of the frequency of persistent firing with or without spikelets was similar. It declined in a nearly linear fashion for a few seconds down to a roughly stable steady state (from 6 to 9 sec poststimulus: 41.09 ± 0.84 Hz). Following this steady state, in most of the recordings, persistent firing terminated suddenly without a further decline in the firing rate (Figure 2(d)).

### 3.2. Persistent Firing Is Expressed by a Physiologically Specific Class of Interneurons

**Figure 3:**
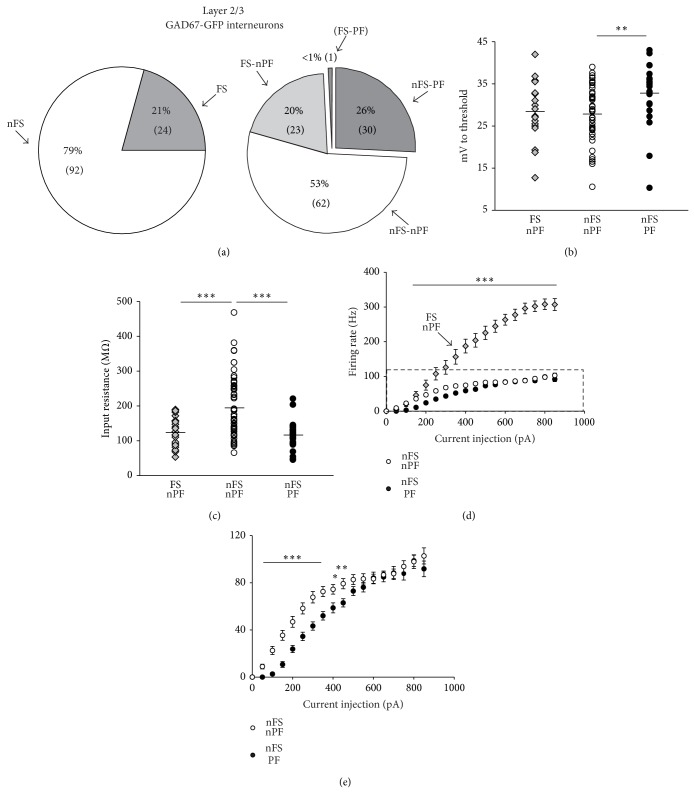
Persistent firing layer 2/3 interneurons showed specific intrinsic electrical properties. (a) The pie chart on the left shows the percentage and number (in braket) of fast-spiking (FS) and non-fast-spiking (nFS) cells of the entire population of recorded layer 2/3 interneurons. The pie chart on the right further subdivides the percentage and number of neurons which express or do not express persistent firing in either fast-spiking or non-fast-spiking interneurons (FS-PF, FS-nPF, nFS-PF, and nFS-nPF) (right). The one and only fast-spiking persistent firing neuron (FS-PF) was not further analysed. The diagrams show (b) Δvoltage (mV difference between resting Vm and spike threshold) and (c) input resistance in the different interneuronal groups (FS nonpersistent firing, FS-nPF; nFS nonpersistent firing, nFS-nPF; nFS persistent firing, nFS-PF). The dots represent single neurons and the horizontal black lines represent the mean of each group. (d) Firing rate versus current injection curve for the three identified interneuronal classes. (e) Magnification of the rectangle dashed area in (d) to emphasize the difference between nFS-nPF and nFS-PF interneurons. Here the curve for FS-nPF was omitted for clarity.

**Figure 4:**
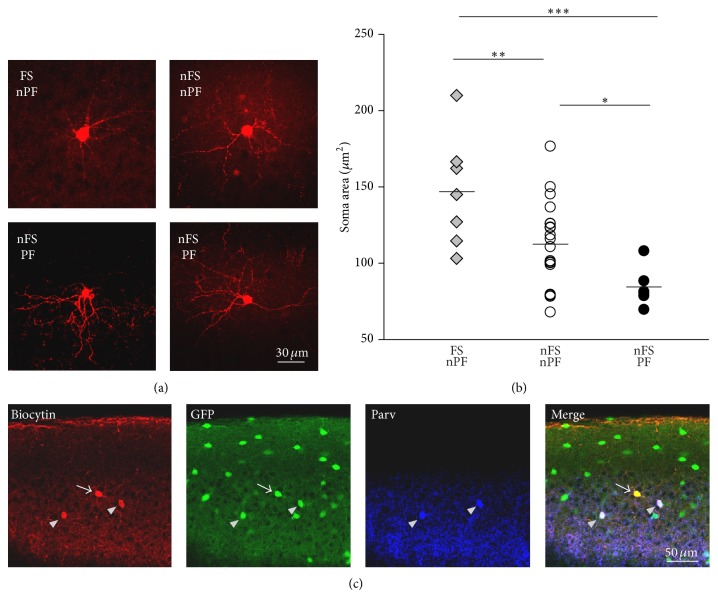
Persistent firing layer 2/3 interneurons showed small soma size and heterologous gap junction coupling. (a) One representative biocytin-filled FS-nPF and nFS-nPF interneuron (top) and two representative biocytin-filled nFS-PF interneurons (bottom). (b) Size of soma in the three electrophysiologically characterized interneuronal classes (FS nonpersistent firing, FS-nPF; nFS nonpersistent firing, nFS-nPF; nFS persistent firing, nFS-PF). (c) Triple immunofluorescence staining for biocytin (red), GFP (green), and parvalbumin (blue). The white arrow points at one representative biocytin-filled nFS-PF neuron. Note the biocytin diffusion from the patched-PF interneuron (white arrow) into two neighboring GFP and parvalbumin-positive interneurons (presumably FS-nPF) (grey arrow heads), suggesting gap junction coupling among these cells.

In our patch-clamp recordings from GAD67-GFP mice we could distinguish between fast-spiking (FS) (20.7%, 24 of 116 neurons) and non-fast-spiking (nFS) interneurons (79.3%, 92 of 116 neurons) (Figure 3(a)) based on the strictly different firing behavior of these cell subtypes upon somatic current injection. Interneurons were considered as FS if upon saturating somatic current injection they could achieve a firing rate of at least 200 Hz (mean maximal firing rate for FS: 301.33 ± 14.73 Hz). The remaining interneurons were considered nFS and their mean maximal firing rate was well below 200 Hz (104.03 ± 2.91 Hz). Interestingly we were able to induce persistent firing in a relatively large portion of nFS interneurons (32.6%, 30 of 92 neurons) but only in one out of 24 FS interneurons (4.2%) (Figure 3(a)). This finding suggests that persistent firing is very unlikely to occur in FS cells or alternatively it may indicate the existence of a very rare but still functionally distinct neuronal type. We decided to not include this single FS, persistent firing cell in further analyses. To examine whether the phenomenon of persistent firing was expressed by a specific functional class of layer 2/3 cortical interneurons we further characterized different physiological and morphological properties in three different populations of recorded interneurons: FS, nonpersistent firing (FS-nPF) (20%, 23 of 116 cells), nFS, nonpersistent firing (nFS-nPF) (53%, 62 of 116 cells), and nFS, persistent firing (nFS-PF) (26%, 30 of 116 cells) (Figure 3(a)). Interestingly, nFS-PF neurons presented a resting Vm significantly more hyperpolarized (*P* < 0.05) and a spike threshold significantly more depolarized (*P* < 0.05) in comparison with nFS-nPF (Table 1). As a consequence the Δvoltage between resting Vm and spike threshold was significantly larger in the nFS-PF than in the nFS-nPF group (*P* < 0.01). This suggests that nFS-PF interneurons require larger Vm depolarization to transit from a resting into an active state. FS interneurons showed intermediate Δvoltage values which did not differ from either of nFS neuronal groups (*P* > 0.05 for both FS versus nFS-nPF and Fs versus nFS-PF) (Figure 3(b), Table 1). nFS-PF interneurons displayed particularly wide somatic APs. The spike half-width in nFS-PF interneurons was not only significantly larger than FS interneurons (*P* < 0.001), which are well described to have very narrow APs [15], but also highly significantly larger than nFS-nPF (*P* < 0.001) (Table 1). It remains to be disclosed if the broad spike width observed in PF interneurons can be attributed to the expression of a specific set of voltage-dependent K^+^ channels with lower kinetics [16] and whether it may have a causal role in the induction of PF. The input resistance did also strongly differ between interneuronal groups. nFS-nPF neurons showed a relatively high input resistance; meanwhile nFS-PF cells displayed significantly lower values (*P* < 0.001), similarly to Fs interneurons (Figure 3(c), Table 1). The reduced input resistance in PF interneurons was not a result of leak currents due to bad recording conditions, since the resting Vm of this neuronal class was not depolarized but even more hyperpolarized than the other neuronal groups. All together, these data suggest that much stronger excitatory inputs are needed to drive nFS-PF interneurons above the spike threshold. To better analyse the relation between neuronal input and output we measured the frequency of action potential firing upon a depolarizing somatic current injection of gradually increasing amplitude. As expected FS interneurons achieved the highest firing rate (in some cells up to 400 Hz) which was highly significantly different from both nFS-nPF and nFS-PF (from 200 to 850 pA *P* < 0.001, Figure 3(d)). Furthermore, nFS-PF interneurons showed a significantly reduced firing compared to nFS-nPF cells at relatively low current injection amplitude (between 150 and 450 pA) (Figure 3(e)). This resulted in a rightward shift of the firing rate versus current injection curve in persistent firing interneurons compared to nFS-nPF (Figure 3(e)). Taken together these findings indicate that interneurons displaying persistent firing possess peculiar membrane properties which make them particularly reluctant to synaptic recruitment. The activation of this neuronal class may require very strong excitatory synaptic inputs. However, neither the frequency nor the amplitude of sEPSCs in nFS-PF were different from nFS-nPF interneurons (Table 1) suggesting a similar functional excitatory connectivity in these two neuronal populations. All together the differences in membrane and firing properties observed between nFS-nPF and nFS-PF cells suggest that the here identified PF interneurons constitute a functional unique neuronal population. Future studies should further investigate the cellular mechanisms responsible for these peculiar intrinsic features. Biocytin-filling further revealed that nFS-PF displayed specific morphological features. nFS-PF neurons always displayed multiple neuronal processes extending from the soma (Figure 4(a), bottom). Interestingly, we also observed differences in the size of soma of the three populations of interneurons. FS interneurons had the largest cell bodies (versus nFS-nPF: *P* < 0.01; versus nFS-PF: *P* < 0.001). In contrast, the soma size of persistent firing interneurons was the smallest (versus nFS-nPF: *P* < 0.05) (Figures 4(a)-4(b)). One additional feature found exclusively in one out of six persistent firing interneurons, but never in nFS-nPF cells (0/21), was the diffusion of biocytin from one recorded PF cell into nearby located interneurons (Figure 4(c), red channel). In this example, the cell pointed by the white arrow was proven to be a PF interneuron. This neuron was the only one from which patch-clamp recordings were performed and therefore the only neuron directly filled with biocytin. It is conceivable that the two additional biocytin-positive neurons (grey arrow heads) were stained indirectly by the diffusion of biocytin from the recorded neuron via gap-junctions [17, 18]. In the second channel in green, the GFP staining confirmed that all three neurons were GAD67-positive inhibitory neurons [10]. The two cells indirectly filled with biocytin (grey arrow heads) but not the recorded one (white arrow) were also found to be immunopositive for parvalbumin (Figure 4(c), blue channel). Since parvalbumin was found to be expressed by FS but never by nFS interneurons (data not shown) it is very likely that the PF, parvalbumin-negative cell, and the two parvalbumin-positive neurons belong to two different neuronal subtypes (nFS-PF and FS-nPF, resp.). This result suggests heterologous gap-junctions coupling between nFS-PF and FS-nPF interneurons.

### 3.3. Layer 1 Interneurons Display Spontaneously Occurring Ectopic Spikes

**Figure 5:**
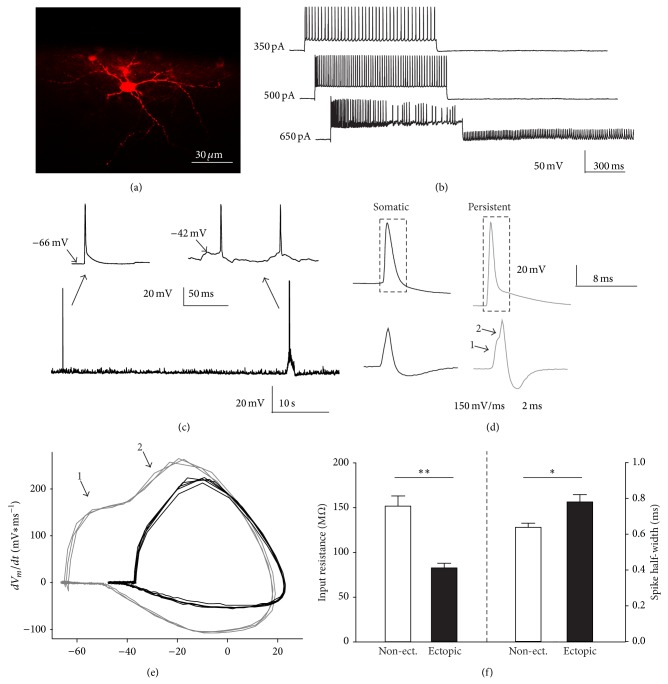
Layer 1 interneurons displayed spontaneous ectopic action potentials (eAPs). (a) One representative biocytin-filled layer 1 interneuron. (b) Representative traces showing a series of depolarizing current steps of increasing amplitude (duration of each step: 1 sec) in the one and only layer 1 interneuron (out of 27 cells) which displayed persistent firing. Note that, in this neuron, persistent firing consisted entirely of spikelets. (c) Voltage trace from one representative layer 1 neuron displaying one spontaneous spike with physiological properties reminescent of ectopically originated action potentials (eAPs). This eAP can be distinguished from the two subsequent “standard” spikes (originated following a spontaneous membrane potential depolarization) due to the different spike waveform and the abrupt, more negative spike threshold. In the inset on top the eAP (left) and the couple of “standard spikes” (right) are streched in time to better visualize their waveforms. (d) Spike waveform of one representative somatic and eAP recorded in a layer 1 interneuron (top) to better distinguish between the two firing “modes.” The derivative of the rectangular dashed area is represented stretched in time in the bottom traces. (e) Phase plot showing the rate of change in membrane potential (dVm/dt) as a function of the membrane potential (Vm) for a few somatic (black traces) and a few ectopic (grey traces) spikes. Similarly to persistent firing, it is possible to observe a biphasic course in the derivative of the spike waveform of the eAP, in (d) (bottom) and (e) (1, 2). (f) Bar graphs showing the mean input resistance (left-axis) and spike half-width (right-axis) for ectopic firing (ectopic firing rate > 0.01 Hz) and nonectopic firing (or ectopic firing at a frequency below 0.01 Hz) layer 1 interneurons.

Finally, we asked whether the phenomenon of somatic APs-induced persistent firing in cortical supragranular layers was exclusively expressed in layer 2/3 or it was also visible in interneurons of cortical layer 1. Recordings of layer 1 neurons were performed from C57BL/6 wild type mice. According to the literature 90–95% of neurons of cortical layer 1 are GABAergic interneurons [19, 20]. Therefore, we presume that the majority of our recordings were obtained from GABAergic cells. Occasionally (5 out of 32 cells) we recorded from neurons which, upon somatic current injection, displayed a firing behavior typical of a pyramidal neuron. In contrast to all other interneurons, these cells displayed a clear spike frequency adaptation, a relatively low maximal firing frequency (ranging between 30 and 40–45 Hz) and a relatively long spike half-width (>1 ms). These neurons were not considered for further analysis. Furthermore we could retrieve six layer 1 biocytin-filled cells and they all showed multipolar somata (Figure 5(a)). We applied, as for layer 2/3 interneurons, a series of one sec-lasting depolarizing current steps of increasing amplitude at 0.1 Hz. From 27 interneurons only one layer 1 interneuron displayed persistent firing following this protocol. In this neuron, persistent firing consisted entirely of spikelets (Figure 5(b)). Although neither a longer nor a more intense protocol was specifically tested, these data suggest that layer 1 interneurons may have a higher threshold for the generation of persistent firing. However, unlike in layer 2/3, roughly half of the recorded layer 1 interneuronal population (44.44%, 12 of 27 cells) occasionally generated spontaneous APs with a waveform and an “apparent” hyperpolarized spike threshold typical of APs originated ectopically, in distal sites of the axon (Figures 5(c)–5(e)). The frequency of these spontaneously occurring ectopic APs (eAPs) varied from cell to cell and differed by several orders of magnitude. In four neurons the rate of eAPs was in the order of 0.001 Hz (2.2 ± 0.33∗10^−3^ Hz) while in another six cells it ranged from 0.01 to 0.1 Hz (4.52 ± 2.60∗10^−2^ Hz). In only two interneurons we observed spontaneous eAPs occurring at high frequency (high rate eAPs, 48.19 and 30.68 Hz). In this neuron eAPs differed from the previously described form of persistent firing only because they did not require somatic current injection to be induced but they were generated without external manipulations following a period of spontaneous activity (some layer 1 interneurons were found to be spontaneously active). We noticed that layer 1 interneurons generating ectopic spikes with a frequency of at least 0.01 Hz displayed a significantly lower input resistance (82.72 ± 5.20 MΩ; *P* < 0.01) and significantly longer spike half-width (0.78 ± 0.04 ms; *P* < 0.05) in comparison with layer 1, nonectopic firing interneurons or interneurons with a very low frequency of ectopic spikes (in the order of 0.001 Hz) (input resistance: 151.76 ± 11.31 MΩ; spike half-width: 0.64 ± 0.02 ms) (Figure 5(f)). This result suggests that ectopic firing layer 1 interneurons share some similarity in membrane and firing properties with persistent firing layer 2/3 interneurons. However, in contrast to layer 2/3, we did not find any significant differences in resting Vm (ectopic: −71.97 ± 1.92 mV; nonectopic/ectopic very low rate: −67.98 ± 1.56 mV) and spike threshold (ectopic: −44.88 ± 3.09 mV; nonectopic/ectopic very low rate: −43.50 ± 1.00 mV) between ectopic and nonectopic (or ectopic at rate <0.01 Hz) cells.

## 4. Discussion

One fundamental property of cortical networks is that during periods of intense neuronal activity the excitatory-inhibitory balance is dynamically maintained within a spatiotemporally defined physiological range by a parallel increase in excitation and inhibition [21], with inhibition following excitation by a few ms [22]. Beyond this fine regulation of neuronal activity on a very short time scale, recent evidence suggests that some cortical inhibitory interneurons can also integrate information about local neuronal activity over a longer time scale and eventually respond with a high frequency firing pattern lasting for tens of seconds [7]. This specific firing behavior has been named persistent firing (PF) and it essentially consisted of atypical action potentials which are, with very high probability, originated at distal axonal sites in absence of synaptic inputs [7]. This aspect has profound implications because it suggests that the axon is not merely a unidirectional relay station but rather a site capable of sensing and integrating information about local neuronal network activity and of relaying such information bidirectionally. The aim of the present study was to characterize the functional profile of the interneuronal cell types expressing the phenomenon of PF.

### 4.1. Which Subclass of Interneurons Expresses PF?

The likelihood of observing the phenomenon of PF strongly varies depending on the subtype of inhibitory interneurons examined. One neuronal subclass expressing very frequently PF is the large population of serotonin 5b receptor-expressing interneurons [7]. Additional studies have also reported that, in the CA1 area of the hippocampus as well as in the piriform cortex, a large portion of persistent firing interneurons are likely to correspond to neurogliaform (NG) cells [8, 9]. These studies suggest that PF interneurons may preferentially express specific neuronal markers and display defined morphological features. Nonetheless, it is still unclear whether some specific physiological properties of GABAergic interneurons can be predictive of the PF behavior and whether they could shed some light on the mechanisms underlying PF induction. Here, we found that supragranular layers cortical interneurons expressing PF presented distinctive membrane properties. PF interneurons were rarely fast-spiking (1 out of 31 PF cells). Furthermore, in comparison with nFS-nPF neurons, they also had a significantly more hyperpolarized resting Vm, more positive spike threshold, wider action potentials, lower input resistance, and smaller soma (Figures 3 and 4). An additional interesting observation was the biocytin diffusion from one recorded nFS-PF cell into two neighboring parvalbumin-expressing interneurons (presumably FS-nPF) (Figure 4(c)). This finding strongly suggested the presence of gap-junctions coupling between two functionally different interneuronal subpopulations. Despite this specific features suggest that PF interneurons form a functionally unique neuronal class, we were not able to assign them to an already existing (already described in the literature) neuronal subtype. For instance, although we found that PF interneurons display some common properties with NG cells, namely, a small soma as well as heterologous gap-junctions (at least in one out of six cases) [23, 24], we failed to evoke PF in layer 1, although this layer is particularly enriched in this cell type [25]. This suggests that the expression of PF may require a specific constellation of anatomical and electrical neuronal features rather than a specific neuronal subclass.

### 4.2. Possible Implications of Spikelets during Persistent Firing

Interestingly, we observed the presence of partial spikes (or spikelets) during some PF episodes. Computer models have shown that partial spikes may represent antidromic action potentials which after backpropagating in the axon fail to invade the somatodendritic compartment of a neuron [7]. Interestingly, partial spikes were mainly present in the initial phase of PF when the firing frequency was very high. Furthermore PF episodes containing partial spikes achieved a higher peak firing frequency. These observations suggest the presence of a gate which allows spikes to back-propagate into the soma only up to a certain frequency. The presence of a sort of low-pass filter, possibly close to the axon initial segment or at the level of the soma, may have important implications for spike timing dependent plasticity-related phenomena which required temporally precise postsynaptic depolarization [26]. Several cellular mechanisms may be responsible for this possible filtering function. In the initial phase of PF some spikes may fail to invade the soma and dendrites because voltage-gated channels in this neuronal compartment may still be partially inactivated due to the prolonged somatic firing (induced by current injection) preceding PF generation. Another possible mechanism could be self-inhibition via autaptic synapses (autapses). Indeed, if PF neurons form inhibitory autapses targeting their own perisomatic region self-inhibition may efficiently limit the spike backpropagation.

### 4.3. Ectopic Firing in Layer 1

Another interesting observation was the difference in the ectopic firing behaviour between layer 2/3 and layer 1 interneurons. Layer 1 interneurons rarely (1 out 27 cells) generated persistent firing in response to repetitive suprathreshold somatic current injections. Nonetheless, a large portion of layer 1 neurons produced spontaneous isolated ectopic spikes during periods of silence. This suggest that, in contrast to the persistent firing behaviour in layer 2/3, the emergence of ectopic firing in this interneuronal population is not strongly influenced by the recent activity of the parent cell. Alternatively, if extracellular signals turn out to be important for the emergence of this peculiar firing behaviour, the concurrent activity of a population of surrounding neurons may be the major drive for the induction of ectopic spikes in layer 1. In support of this hypothesis a relatively high number of spontaneously active cells were found in the outmost cortical layer (44%, 12 of 27 layer 1 interneurons). This hypothetical scenario suggests that ectopically firing in layer 1 may convey information about the recent activity in a local neuronal population rather than in single neurons.

### 4.4. Hypothesis on the Cellular Induction Mechanisms

Concerning the mechanisms underlying PF generation little is known. It has been reported that (1) it requires hundreds of somatic action potentials either in the parental or in a neighboring, presumably gap-junction coupled neuron, (2) it does not require chemical synaptic transmission [7], and (3) it is modulated by extracellular Ca^2+^ level. Specifically, low level of Ca^2+^ (<1 mM) was found to prolong the duration of PF [27]. Although our study did not aim to disclose the induction mechanisms we put forward the hypothesis that changes in extracellular K^+^ level could have a key role in PF generation. Specifically, retrograde persistent spikes may be induced by a local increase in extracellular K^+^which could transiently depolarize axonal terminals above the spike threshold [28]. This scenario is plausible since the induction of PF always required a large number and relatively high frequency of APs (Figure 1) and intense-prolonged neuronal activity is well documented to cause a transient rise in K^+^concentration in extracellular microdomains [29, 30]. The decline in extracellular K^+^level following high neuronal activity is also suitable to explain the temporal dynamic of PF. We and others showed that PF outlasts the stimulus duration for few seconds in the cortex (Figure 2) or for tens of seconds to minutes in the hippocampus [7]. Remarkably, glia cells do require a similar time to restore normal level of extracellular K^+^. Indeed the main glial K^+^ buffering systems [29] are also operating in the range of tens of seconds [28, 29].

Based on this hypothesis PF interneurons should display biophysical properties favoring the accumulation of extracellular K^+^. However, the physiological properties which distinguished PF from nPF interneurons, such as non-fast-spiking phenotype, hyperpolarized resting Vm, and lower input resistance, cannot directly explain a preferential K^+^ accumulation in extracellular microdomains. Nonetheless, the “K^+^ hypothesis” may explain why NG cells have a high propensity to generate persistent ectopic APs. Indeed, NG cells are characterized by dense axonal arborizations [31], an anatomical feature which is likely to favor a robust and rapid rise of K^+^ in a very restricted extracellular volume. The “K^+^ hypothesis” may also explain why we failed to induce PF in layer 1 interneurons. The very low cellular density and the consequent relative large extracellular volume in this outmost cortical layer may indeed prevent robust accumulation of extracellular ions following activity of one single neuron. Alternatively, intense activity in a population of neighbouring neurons may be required to cause a transient increase in extracellular K^+^ sufficient to cause the sparse ectopic firing observed in layer 1. This hypothetic scenario is supported by the fact that a large portion of interneurons in layer 1 were found to be spontaneously active. Future studies by performing localized application of varying concentration of K^+^ at different points along the axons of PF neurons should further evaluate the role of K^+^ and glial K^+^ buffering in PF induction and maintenance.

### 4.5. Does Persistent Firing Occur in Physiological or Pathophysiological Contexts?

As previously discussed, PF interneurons displayed membrane properties (low input resistance, large Δvoltage between resting Vm and spike threshold, Figure 3) tending to minimize their recruitment during physiological network activity. The unaltered sEPSCs frequency as well as amplitude compared to nFS-nPF interneurons (Table 1) suggests that particularly strong or numerous excitatory synaptic inputs are unlikely to compensate for the low intrinsic excitability. Therefore, the here reported distinctive physiological properties characterizing PF interneurons do not promote but rather dampen action potential firing. It has been reported that PF can also be initiated by firing patterns naturally occurring* in vivo* [7]. Nonetheless, in light of the current findings, one may wonder under which circumstances can the interneuronal subtype displaying PF fire many APs at relatively high frequency (necessary condition to induce persistent firing). To achieve this scenario, PF interneurons would require a strong and sustained depolarization above the spike threshold. Excitatory synaptic inputs are most likely not sufficient to provide such condition unless the cortical network is in pathological, hyperexcitable state. In this regard, it has already been proposed that PF in cortical interneurons may have a role in dampening excessive excitation during epileptiform activity [8]. If this is the case, the low intrinsic excitability of PF expressing interneurons (Figure 3) may be important to guarantee that this protective mechanism will be engaged only in pathological circumstances of abnormal neuronal activity.

Beside this plausible scenario, PF interneurons could also be recruited in absence of hyperexcitability through electrical synapses. In particular, they could be stimulated through heterologous gap junctions by other interneuronal subclasses, such as parvalbumin-expressing interneurons (Figure 4(c)) which are well known to fire at high frequency under physiological condition [32]. Future studies by performing a selective optogenetic activation of parvalbumin-expressing neurons may provide an answer to this question. Since parvalbumin-positive interneurons are also well known to be implicated in the generation of high frequency oscillations in the cortex [33] it is tempting to speculate that the recruitment of PF interneurons via heterologous electrical synapses may serve to promote the generation or the spreading of gamma oscillations.

Although this hypothesis is purely speculative, it is curious to observe that the frequency of persistent firing varies from around 40 to 90 Hz (Figure 2) which roughly corresponds to the gamma band range [34].

## Figures and Tables

**Table 1:** Membrane and synaptic properties of fast-spiking nonpersistent firing (FS-nPF), non-fast-spiking nonpersistent firing (nFS-nPF), and non-fast-spiking persistent firing (nFS-PF) layer 2/3 interneurons. The column *P* 1–3 represents the *P* values between FS-nPF (1) and nFS-PF (3); meanwhile the column *P* 2-3 represents the *P* values between nFS-nPF (2) and nFS-PF (3).

Layer 2/3	FS-nPF (1)	nFS-nPF (2)	nFS-PF (3)	*P* 1–3	*P* 2-3
Resting Vm	−69.45 ± 1.14 mV (19)	−68.51 ± 0.78 mV (51)	−71.10 ± 0.96 mV (25)	>0.05	**0.049**
Spike threshold	−40.71 ± 1.04 mV	−41.15 ± 0.77 mV	−38.52 ± 0.95 mV	>0.05	**0.042**
mV to threshold	28.44 ± 1.64 mV	27.84 ± 1.01 mV	32.80 ± 1.50 mV	>0.05	**0.008**
Input resistance	123.39 ± 8.69 MΩ	194.43 ± 11.03 MΩ	116.33 ± 7.05 MΩ	>0.05	**<0.001**
Spike half-width	0.36 ± 0.01 ms	0.60 ± 0.01 ms	0.68 ± 0.02 ms	**<0.001**	**<0.001**
sEPSCs frequency	—	4.84 ± 0.52 Hz (25)	5.78 ± 0.75 Hz (8)	—	>0.05
sEPSCs amplitude	—	14.15 ± 0.99 pA (25)	13.22 ± 1.89 pA (8)	—	>0.05
Soma size	146.90 ± 12.73 *μ*m^2^ (7)	112.50 ± 5.85 *μ*m^2^ (21)	84.44 ± 4.87 *μ*m^2^ (6)	**<0.001**	**0.037**
